# What is the gap in activity and participation between people with disability and the general population in Taiwan?

**DOI:** 10.1186/s12939-017-0628-5

**Published:** 2017-08-01

**Authors:** Tzu-Ying Chiu, Chia-Feng Yen, Reuben Escorpizo, Wen-Chou Chi, Tsan-Hon Liou, Hua-Fang Liao, Cheng-Hsiu Chou, Wen-Hui Fang

**Affiliations:** 1 Institute of Health Policy and Management, National Taiwan University, Taipei, Taiwan; 2 Department of Public Health, Tzu Chi University, No.701, Sec. 3, Zhongyang Rd, Hualien, 970 Taiwan, Republic of China; 3 Department of Rehabilitation and Movement Science, College of Nursing and Health Sciences, University of Vermont, Burlington, USA; 4 Swiss Paraplegic Research, Nottwil, Switzerland; 5 School of Occupational Therapy, Chung Shan Medical University, Taichung, Taiwan; 6 Department of Physical Medicine and Rehabilitation, Shuang Ho Hospital, Taipei Medical University, Taipei, Taiwan; 7 Graduate Institute of Injury Prevention and Control, Taipei Medical University, Taipei, Taiwan; 8 Department of Physical Medicine and Rehabilitation, School of Medicine, College of Medicine, Taipei Medical University, Taipei, 11031 Taiwan; 9 School and Graduate Institute of Physical Therapy, National Taiwan University, Taipei, Taiwan; 10 Departments of Family Medicine, Hualien Armed Forces General Hospital, Hualien, Taiwan; 11 Institute of Health Administration, Tri-Service General Hospital, Taipei, Taiwan

**Keywords:** Activity and participation (AP), Whodas 2.0, Adults with disability, Population norm, Normative value

## Abstract

**Background:**

In 2010, the World Health Organization Disability Assessment Schedule 2.0 (WHODAS 2.0) was developed, based on the concept of the International Classification of Functioning, Disability and Health (ICF). The ICF provides a common language and framework for health and health-related status and attempts to integrate the biopsychosocial model as a multidimensional perspective in understanding functioning. Activities and participation (AP) is one salient component of the ICF refers to the execution of a task by an individual, and how such tasks are involved in their daily life. It is essential to examine the gap between the general adult population and adults with disabilities. This gap may be attributed to health status, personal factors, and natural and social environments, which include social and health services and policies. The purposes: (1) To develop a normative activity and participation (AP) value for the adult population and people with disabilities; and (2) to compare the gap in AP normative values between the two groups in Taiwan.

**Methods:**

We use the WHODAS 2.0 to survey and develop a normative AP value for the general adult population, and used secondary data from National Disability Eligibility Determination System (NDEDS) of Taiwan to describe the AP functioning distribution of adult with disability. There were 1100 participants, selected by stratified proportional sampling from two cities. There were also 144,850 participants who were adults with disability, selected from the secondary database in Taiwan.

**Results and conclusions:**

The AP curve for the disabled population increased rapidly at the beginning. The summary score was 13.21 in the performance at 90 percentile for the general population and 82.61 score for disabled adults that the similar gap in every domain, its means that there are significant functioning difference and health equality in general adults population and adults with disabilities. This presents a substantial challenge for both the government and the whole population of Taiwan, to begin considering how to reduce the gap in AP functioning and promote equality for people with disabilities, using social welfare policy. It is important to make sure disabled people have the same rights to be included in society as anybody else and better access to things in all areas of life that are according to Convention on the Rights of Persons with Disabilities (CRPD).

## Background

Addressing individual problems in functioning is becoming more common in epidemiological studies. There are several measurements of health status and health-related quality of life. These include the Short Form Health Survey (SF-36, SF-12), the World Health Organization (WHO) Quality of Life-BREF (QOL-BREF), Activities of daily living (ADL), the Instrumental Activities of Daily Living Scale (IADL), and the EuroQoL-5 Dimension Questionnaire (EQ-5D) [1–12]. Historically, country-specific normative values were developed for most of these, based on different data collection methods.

In 2010, the World Health Organization Disability Assessment Schedule 2.0 (WHODAS 2.0) was developed, based on the concept of the International Classification of Functioning, Disability and Health (ICF) [13]. The ICF provides a standard language and framework for health and health-related states [14–16] and attempts to integrate the biopsychosocial model as a multidimensional perspective in understanding disability. Activities and participation (AP) is one salient component of the ICF, which distinguishes it from the traditional biomedical model. The concept of AP refers to the execution of a task by an individual, and how such tasks are involved in their daily life [14].

WHODAS 2.0 is an established measurement instrument that can capture the AP status of an individual. As a measurement instrument, it has sound theoretical underpinnings and psychometric properties. Research has also shown that the WHODAS 2.0 can be used to assess health and disability levels in the general adult population using surveys, and to measure the clinical effectiveness of and productivity gains resulting from interventions [13].

So far, there has been only one study of WHODAS 2.0 norms, which was implemented by the European Union [13, 17]. This was the WHO Multi-Country Survey Study on Health and Responsiveness 2000–2001 (MCSS), and it included 61,175 household cases from 10 countries (Colombia, Egypt, Georgia, India, Indonesia, Islamic Republic of Iran, Lebanon, Mexico, Nigeria, Singapore, Slovakia, Syria and Turkey) [13, 17]. This study was based on different methods of data collection. In most cases, the WHODAS 2.0 was administered face-to-face and the results indicated that that data collection method was important for population AP normative value development. Moreover, the study also demonstrated the application of WHODAS 2.0 in many countries. However, an AP norm for populations in Asia and Greater China is still lacking.

The People with Disabilities Rights Protection Act has regulated disability evaluation to enhance the social participation of adults with disabilities in Taiwan [18]. In 2012, the WHODAS 2.0 was included as part of an evaluation instrument used in the adult assessment, conducted under the NDEDS system in Taiwan, with the aim of understanding and measuring the limitations and restrictions on AP for adults with disabilities [15, 16, 19]. It is essential to understand the functioning problems of adults with disabilities and to examine the gap between the general adult population and adults with disabilities in Taiwan. This gap may be attributed to health status, personal factors, and natural and social environments, which include social and health services and policies. Understanding this gap will allow the government of Taiwan to appropriately allocate specific resources and to develop more concrete plans. A normative value of AP can serve as a benchmark to interpret disability across different groups of individuals.

The aims of present study are: (1) to develop a normative AP value for the adult population in Taiwan; (2) to describe the distribution of that value; and (3) to identify and compare the gap in normative AP values between the general adult population and adults with disabilities in Taiwan. This study is the first to use the face-to-face interview method to gauge AP, the assessment of which has been a significant factor in designing the Taiwanese disability social welfare services and services delivery system.

## Methods

To develop the normative AP value, we performed a cross-sectional survey from August 2013 to July 2014. The sampling design was stratified, with proportional sampling of two cities in Taiwan: Taipei City (the capital of Taiwan), and Hualien County. These two cities were representative of urban and rural areas, respectively. According to Taiwan’s National Health Research Institutes [20], urban and rural are defined based on the following variables: population density (people/km^2^), proportion of people in the population with college-level education or higher, proportion of the population over the age of 65 years, proportion of the population in agricultural work, and number of physicians per 100,000 people. To categorize the locations in the current study, we operationally defined Taipei City as urban and Hualien County as rural.

To determine the AP level of adults with disability, we used secondary data from the NDEDS. We used the seventh version of the database, which was the latest version of the assessment, based on WHODAS 2.0 and the same instrument as was used in the general population portion of the study. This data collection period was November 2013 to January 2015. We defined adults as people aged 18 years and above.

### Participants and data collection

#### Normative value population and participants

In December 2013, the population of Taipei was 2,172,312 and of Hualien County were 273,915. Our samples were selected by stratified proportional sampling according to sex and age group in the two cities. The sampling frame was selected according to an up-to-date registry from the Ministry of Interior (MOI) of Taiwan taken in 2013 [21]. We surveyed 305 people in Hualien and 804 in Taipei. There was no statistical difference in sex, age, or the proportion of people with a disability between the two groups. Thus our general adult samples were reasonably representative of the rural and urban populations of Taiwan (Table 1).

**Table 1:** Sample characteristics

Variable		Urban^a^		χ^2^ (*P* value)	Rural^b^		χ^2^ (*P* value)	All		χ^2^ (*P* value)
		Population n(%)	Sample n(%)		Population n(%)	Sample n(%)		Population n(%)	Sample n(%)	
Sex		2,172,312	797	0.04 (0.84)	273,914	303	2.33(0.13)	**2,446,496**	**1100**	0.30(0.59)
	Male	1,027,273(47.29)	374(46.9)		140,384(51.25)	142(46.9)		1,167,657(47.73)	516(46.9)	
	Female	1,145,309(52.71)	423(53.1)		133,530(48.75)	161(53.1)		1,278,839(52.27)	584(53.1)	
Age		2,172,312	797	6.46 (0.60)	273,915	303	5.05(0.75)	**2,446,227**	**1100**	9.12(0.33)
	18–19	63,794(2.94)	12(1.5)		9639(3.52)	5(1.7)		73,433(3)	17(1.5)	
	20–29	331,131(15.24)	120(15.1)		47,643(17.39)	56(18.5)		378,774(15.48)	176(16)	
	30–39	427,287(19.67)	164(20.6)		52,062(19.01)	60(19.8)		479,349(19.6)	224(20.4)	
	40–49	432,506(19.91)	163(20.5)		52,983(19.34)	56(18.5)		485,489(19.85)	219(19.9)	
	50–59	419,719(19.32)	153(19.2)		50,812(18.55)	52(17.2)		470,531(19.23)	205(18.6)	
	60–69	255,692(11.77)	99(12.4)		30,059(10.97)	37(12.2)		285,751(11.68)	136(12.4)	
	70–79	146,242(6.73)	51(6.4)		19,222(7.02)	25(8.3)		165,464(6.67)	76(6.9)	
	80–89	81,752(3.76)	30(3.8)		10,049(3.67)	11(3.6)		91,801(3.75)	41(3.7)	
	≧90	14,189(0.65)	5(0.6)		1446(0.53)	1(0.3)		15,635(0.64)	6(0.5)	
People with Disability										
	No	96.12%	96.2%	0.10(0.92)	91.27%	95%	3.36(0.07)	95.8%	95.9%	0.01(0.93)
	Yes	3.88%	3.8%		7.83%	5%		4.2%	4.1%	

^a^The adult population in urban areas based on the 2013 statistics of the Ministry of the Interior in Taiwan

^b^The adult population in rural areas based on the 2013 statistics of the Ministry of the Interior in Taiwan

The initial number of participants for the population norm evaluation was 1109. We excluded people aged less than 18.0 years (*n* = 1) and those with ≥50% of items missing in each domain (*n* = 8) [19, 22, 23]. This gave us a final sample of 1100 participants who were older than 18 years and community-dwelling individuals from Taipei City or Hualien County. The data were collected face-to-face by interviewers who were qualified after professional training courses. The study was approved by the Buddhist Tzu Chi General Hospital Research Ethics Committee (IRB102–24).

#### Adults with disability

At the time of the present study, there were a total of 144,850 adults in the whole of Taiwan who qualified as disabled in the NDEDS. The NDEDS is a social security system used to identify those who qualify for subsidies and in-kind services in Taiwan. Individuals in the system were evaluated via face-to-face interview by physicians and an occupational therapist, physical therapist, social worker, clinical psychologist, or nurse practitioner in the hospitals. The databank included a record of demographic characteristics (including personal factors), evaluations of the individual’s body function and body structures, AP functioning (from the WHODAS 2.0), and some environmental conditions.

### Instruments

The present study was conducted using the traditional Chinese version of WHODAS 2.0 which is the one part of the Functioning Disability Evaluation Scale adult version (FUNDES-Adult) in NDEDS. The traditional Chinese version of WHODAS 2.0 was developed in 2014, and includes bilingual translation, examination of internal consistency, test-retest, content validity, concurrent validity and construct validity [23] which was been used in seventh version of FUNDES-Adult in NDEDS. The aims of these assessments were to measure AP in daily life over the previous 30 days [19, 23] in two dimensions and six domains. The two dimensions were “performance” and “capability”. Performance refers to the extent of restriction on participation in daily life and the qualifier of performance is described as what an individual does in his or her current environment. Since the current environment always includes the overall societal context, performance can also be understood as “involvement in a life situation” or “the lived experience” of people in their actual context. The capability refers to the extent of restriction on participation in a real environment without assisting by any assistive device or persons. These dimensions therefore capture the extent of difficulty in daily life without the use of an assistive device or another person’s help [19].

The six domains are (1) cognition (6 items): assesses communication and thinking activities, including concentrating, remembering, problem solving, learning and communicating; (2) getting around (5 items): assesses activities such as standing, moving around inside the home, getting out of the home and walking long distances; (3) self-care (4 items): assesses hygiene, dressing, eating and staying alone; (4) getting along with people (5 items): assesses interactions with other people and difficulties that may be encountered in this domain due to health conditions; (5) life activities (household and school/work, 8 items): assesses difficulty with day-to-day activities (i.e. those that people do on most days, including those associated with domestic responsibilities, leisure, work, and school), and (6) participation (8 items): assesses social dimensions, such as community activities; barriers and hindrances in the world around the respondent, and problems with other issues, such as maintaining personal dignity. The possible responses to each item are 1: no difficulty, 2: mild difficulty, 3: moderate difficulty, 4: severe difficulty, and 5: extreme difficulty. The grades of all items in six domains were transferred to domain scores and summary scores. The scores syntax was calculated by WHODAS 2.0 manual and the scoring methods were based on the item-response-theory (IRT), so it could be compare with different population [13]. The total score ranged from 0 to 100 in every domain, with a higher score indicating higher limitation/restriction in daily life.

### Statistical analysis

All data were analyzed using IBM SPSS 20.0, and significance was assumed at a *p*-value of 0.05. We investigated the distribution of participant characteristics using descriptive analysis and the range of domain scores using the ceiling and floor effects. The ceiling effect refers to the proportion of participants who scored 100 (extremely high limitation/restriction), and conversely the floor effect is the proportion of participants who scored 0 (no limitation/restriction). Data were excluded if more than 50% of the items were missing in each domain (*n* = 8); otherwise we use the domain’s mean of every participant (the mean score of themselves in every domain) as imputation based on the WHODAS 2.0 manual [19, 22, 23]. The imputed rate was 0.2%–17.4% among six domains in general population of the participation and capability dimensions and 4.4%–51.6% in disabled population. Furthermore, the highest missing item was “item 4.5 sexual activity”, and this result was consistent with the previous literatures in the world [19, 24–26].

## Results

### Characteristics of general adult population and adults with disabilities

The majority of the general adult population was female (53.1%) and the majority of adults with disabilities were male (53.8%; Table 2). The average age of adults with disabilities was greater than the general adult population. Most of the general adult population undertook paid work (55.7%), compared to the 9.8% of adults with disabilities. In the general adult population, 2.8% were unemployed, compared 54.9% of adults with disabilities. There were 4.1% of the general adult population with disability (Table 1) and the proportions of the general adult population with different levels of disability were 29.5% (mild), 47.7% (moderate), 13.6% (severe), and 9.1% (profound), relative to 39.5%, 31.3%, 16.7%, and 12.5%, respectively, for the adults with disabilities (Table 2).

**Table 2:** The demographic characteristics of samples and people with disabilities

Variable	Samples (*n* = 1100)		People with disabilities (*n* = 144,850)	
	n	%	n	%
Gender	1100		144,850	
Male	516	46.9	77,931	53.8
Female	584	53.1	66,919	46.2
Age	46.9±17.15		60.75 ± 18.28	
18–19	17	1.5	1934	1.3
20–29	176	16.0	6472	4.5
30–39	224	20.4	12,125	8.4
40–49	219	19.9	19,174	13.2
50–59	205	18.6	26,651	18.4
60–69	136	12.4	25,733	17.8
70–79	76	6.9	26,413	18.2
80–89	41	3.7	22,318	15.4
≧90	6	0.5	4030	2.8
Working status	1093		144,850	
Paid-work	610	55.7	14,223	9.8
Self-employed	96	8.8	2515	1.7
Unemployed	31	2.8	79,522	54.9
Homemaker	136	12.5	10,007	6.9
Retired	170	15.5	37,044	25.6
Students	61	5.6	1539	1.1
Disability certification	1088		144,850	-
No	1043	95.9	0	0
Yes	45	4.1	144,850	100
Severity of Disability	44		144,850	
Mild	13	29.5	57,257	39.5
Moderate	21	47.7	45,309	31.3
Severe	6	13.6	24,173	16.7
Profound	4	9.1	18,111	12.5

### WHODAS 2.0 scores of the general adult population and adults with disabilities

Average performance scores differed substantially between general adults and adults with disabilities. The average performance score of general adults was 4.14 ± 9.21, and the domains in which they experienced the most difficulty were participation (6.08 ± 12.46), life activities: household (4.52 ± 13.32) and cognition (4.05 ± 10.42). The average performance score for adults with disabilities was 45.10 ± 25.54, and their most difficult domains were life activities: work and school tasks (82.33 ± 34.51), life activities: household (53.08 ± 39.96) and getting along (49.11 ± 34.15).

The median in all domains for the general adult sample was 0, and more than 57.6% of these adults had summary scores of 0 in every domain. The ceiling effect in every domain was less than 0.5% for both dimensions. These findings indicate that most adults in Taiwan have no difficulties or limitations in AP functioning with respect to either performance or capability (Table 3). In contrast, for adults with disabilities, the median was significantly higher than for the general adult population, especially in the domain of life activity: work and school tasks. The ceiling effect of adults with disabilities ranged between 1.7 and 77.6%, and scores in the capability dimension were significantly higher than those in the performance dimension (*p* < 0.05; Table 3).

**Table 3:** The WHODAS 2.0 scores of the general adult population and the adults with disabilities in Taiwan (Do = domain)

General adults – performance (*n* = 1100)	Do1. cognition	Do2. mobility	Do3. self-care	Do4. getting along	Do5–1. life activities: household	Do5–2. life activities: work and school tasks	Do6. participation	Summary score
Mean ± SD	4.05 ± 10.42	4.00 ± 11.80	1.62 ± 7.71	3.34 ± 10.38	4.52 ± 13.32	2.60 ± 9.21	6.08 ± 12.46	4.14 ± 9.21
Paired t-test^¥^	5.20***	4.28***	4.24***	3.24**	4.78***	3.98***	5.48***	6.22***
Median	0	0	0	0	0	0	0	0
Range	0–100	0–100	0–90	0–100	0–100	0–100	0–100	0–99.06
Floor effect^a^ (n/%)	833(75.7)	919(82.9)	1028(93.5)	944(85.8)	935(85)	833(88.2)	728(66.2)	634(57.6)
Ceiling effect^a^ (n/%)	2(0.2)	1(0.1)	0	1(0.1)	4(0.4)	2(0.2)	1(0.1)	0
General adults – Capability (*n* = 1100)								
Mean ± SD	4.66 ± 12.07	4.51 ± 13.36	2.05 ± 9.72	3.84 ± 12.06	5.44 ± 15.98	2.98 ± 10.37	6.58 ± 13.44	4.71 ± 10.66
Median	0	0	0	0	0	0	0	0
Range	0–100	0–100	0–100	0–100	0–100	0–100	0–100	0–100
Floor effect^a^ (n/%)	823(74.8)	907(82.5)	1020(92.7)	938(85.3)	923(83.9)	823(87.3)	719(65.4)	627(56.0)
Ceiling effect^a^ (n/%)	3(0.3)	1(0.1)	1(0.1)	2(0.2)	6(0.5)	2(0.2)	1(0.1)	1(0.1)
Adults with disabilities – Performance (*n* = 144,850)								
Mean ± SD	39.77 ± 31.75	38.71 ± 34.20	25.24 ± 30.59	49.11 ± 34.15	53.08 ± 39.96	82.33 ± 34.51	43.23 ± 26.53	45.10 ± 25.54
Paired t-test ^¥^	153.87***	217.91***	215.49***	102.09***	141.36***	49.47***	154.99***	264.43***
Median	35	31.25	10	50	50	100	41.67	42.45
Range	0–100	0–100	0–100	0–100	0–100	0–100	0–100	0–100
Floor effect^a^ (n/%)	19,124(13.2)	33,140(22.9)	58,198(40.2)	23,692(16.4)	34,425(23.8)	6667(7.6)	5949(4.1)	1198(0.8)
Ceiling effect^a^ (n/%)	12,282(8.5)	13,893(9.6)	7922(5.5)	19,192(13.2)	42,978(29.7)	68,458(77.6)	5218(3.6)	2415(1.7)
Adults with disabilities – Capability (*n* = 144,850)								
Mean ± SD	43.37 ± 32.26	48.15 ± 38.92	39.85 ± 38.56	51.66 ± 34.49	62.27 ± 37.81	83.65 ± 32.87	47.01 ± 27.39	51.12 ± 27.46
Median	40	43.75	30	50	70	100	45.83	49.06
Range	0–100	0–100	0–100	0–100	0–100	0–100	0–100	0–100
Floor effect^a^ (n/%)	15,841(10.9)	28,782(19.9)	44,495(30.7)	21,614(14.9)	21,021(14.5)	5270(6)	4533(3.1)	782(0.5)
Ceiling effect^a^ (n/%)	14,696(10.1)	33,360(23)	23,734(16.4)	22,820(15.8)	53,377(36.8)	68,910(47.6)	7540(5.2)	4336(3)

^¥^There are significant differences between performance and capability in both groups, *P* < 0.001***; *P* < 0.01**; *P* < 0.05*

^a^The ceiling effect is the proportion of 100 scores and the floor effect is the proportion of 0 score

According to the National Health Insurance (NHI) Law in Taiwan, the NHI will supply a general health checkup every year for people aged over 65 years and one checkup every three years for people between 40 and 64 years of age. Because of this, we compared these three age groups (Table 4). In the general adult population, for the different age and sex groups, there was a small change below the 95 percentile in all age groups; they all scored less than 20.75 in the performance dimension. These findings indicate that almost 5% of people in Taiwan have clear difficulties in AP. In both dimensions, a large majority of adults in the 18–39 and 40–64 age groups who were having problems in daily life were males, contrary to the older age group (Table 4).

**Table 4:** The WHODAS 2.0 norms score of samples by gender and age group

Performance	All	Male				Female			
		18–39	40–64	≥65	All Male	18–39	40–64	≥65	All Female
n	1100	207	231	78	516	210	277	97	584
Mean ± SD	4.14 ± 9.21	3.99 ± 7.81	3.46 ± 7.98	6.55 ± 12.69	4.14 ± 8.83	3.31 ± 6.16	2.8 ± 6.59	9.81 ± 17.52	4.15 ± 9.55
Median	0	0	0	1.09	0	0	0	2.17	0.00
Range	0–99.06	0–66.04	0–51.89	0–72.83	0–72.83	0–30.19	0–37.74	0–99.06	0–99.06
30 percentile	0.00	0.00	0.00	0.00	0.00	0.00	0.00	0.94^b^	0.00
40 percentile	0.00	0.00	0.00	0.00	0.00	0.00	0.00	1.09^b^	0.00
50 percentile	0.00	0.00	0.00	1.09	0.00	0.00	0.00	2.17^b^	0.00
60 percentile	0.94	1.70^a^	0.00	2.83	0.94	0.94	0.00	4.35^b^	0.94
70 percentile	2.83	4.34^a^	1.89^a^	3.77	3.26^a^	2.83	.94	7.21^b^	2.17
80 percentile	5.66	6.60^a^	4.57^a^	13.04	6.18^a^	6.35	3.77	16.14^b^	5.66
85 percentile	8.49	9.43^a^	7.55^a^	15.38	9.43^a^	7.88	5.66	21.30^b^	8.49
90 percentile	13.21	13.40^a^	10.38^a^	21.92	13.48^a^	12.26	8.68	31.14^b^	13.21
95 percentile	20.75	16.04	20.75^a^	34.06	20.75	16.98^b^	18.96	50.19^b^	21.73^b^
100 percentile	99.06	66.04^a^	51.89^a^	72.83	72.83	30.19	37.74	99.06^b^	99.06^b^
Capability	All	Male				Female			
		18–39	40–64	≥65	All Male	18–39	40–64	≥65	All Female
n	1100	207	231	78	516	210	277	97	584
Mean ± SD	4.71 ± 10.66	4.61 ± 8.6	3.6 ± 8.91	7.64 ± 13.8	4.62 ± 9.77	3.77 ± 7.19	3 ± 7.77	12.09 ± 20.9	4.79 ± 11.39
Median	0	0	0	1.09	0.00	0	0	2.17	0.00
Range	0–100	0–64.15	0–73.58	0–70.65	0–73.58	0–41.51	0–77.36	0–100	0–100
30 percentile	0.00	0.00	0.00	0.00	0.00	0.00	0.00	.94^b^	0.00
40 percentile	0.00	0.00	0.00	0.00	0.00	0.00	0.00	1.09^b^	0.00
50 percentile	0.00	0.00	0.00	1.09	0.00	0.00	0.00	2.17^b^	0.00
60 percentile	0.94	1.89^a^	0.00	2.83	0.94	0.94	0.00	5.43^b^	0.94
70 percentile	3.26	4.72^a^	1.89^a^	4.35	3.77^a^	2.83	0.94	8.7^b^	2.83
80 percentile	6.60	7.55^a^	4.72^a^	15.43	6.60^a^	6.60	3.77	17.58^b^	6.52
85 percentile	9.43	12.26^a^	7.55^a^	18.80	10.38^a^	8.49	5.66	28.26^b^	8.49
90 percentile	15.09	15.09^a^	10.38^a^	23.62	15.09^a^	13.21	8.68	44.13^b^	14.14
95 percentile	22.64	19.43	20.75^a^	40.71	20.90	20.24^b^	18.96	65.76^b^	24.53^b^
100 percentile	100.00	64.15^a^	73.58	70.65	73.58	41.51	77.36^b^	100^b^	100.00^b^

^a^Male Scores > Female Scores; ^b^Female Scores > Male Scores

### Comparison of different population AP normative values: The gap in AP functioning

**Fig. 1:**
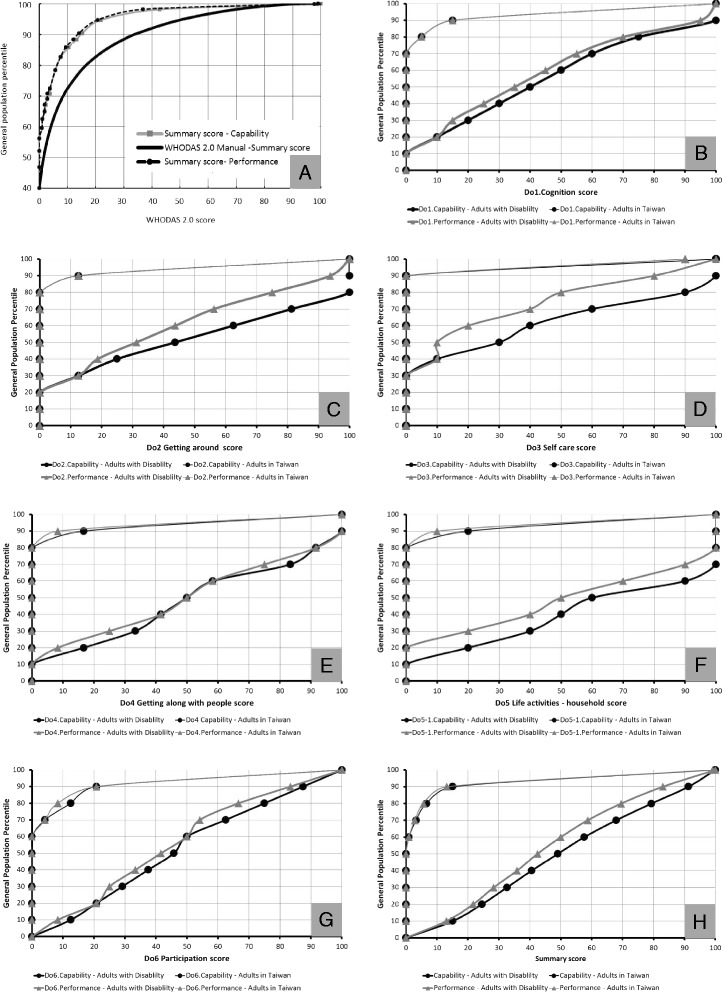
The functioning problem difference between adults population and adults with disabilities

There were similar patterns in the AP functioning norms for performance and capability from our study and the scores from the multiple-countries survey in the WHODAS 2.0 manual for summary scores (Fig. 1a) [13]. The lower the WHODAS 2.0 score, the greater the proportion of the general population that achieved that score. The performance and capability curves in our study were also similar [13]. Moreover, in both dimensions, the percentile at the start point (Fig. 1a) indicates that nearly 58% of the population had no difficulties or limitations in daily life. This is higher than the WHO reference score curve, which indicates 40%, and implies that the general adult population of Taiwan reported fewer difficulties and limitations in daily life and better functioning than populations from the other countries referenced in the WHODAS 2.0 manual.

On the other hand, in the 90th percentile the scores in these two studies were 14 and 35 points, respectively, and in the 95th percentile were 20 and 50 points, respectively. These findings indicate that below the 90th percentile, the gap between the scores recorded in the two studies expanded as the percentile increased, although performance was similar above the 90th percentile (Fig. 1a).

We also compared our data on the general adult population with data and adults with disabilities in Taiwan, and we found that there were differences between these datasets in every domain and in both dimensions (Fig. 1b-h). Most people in Taiwan reported no problems or limitations in daily life (the 60th percentile scored 0), whereas even the lowest percentile of adults with disabilities reported slight difficulties (Fig. 1h). These two curves differ considerably in domain 5: life activities (the largest area of difference, Fig. 1f), domain 4: getting along with people (the second-largest area of difference, Fig. 1e), domain 2: getting around (the third-largest area of difference, Fig. 1c), summary score (the fourth-largest area of difference, Fig. 1h), domain 3: self-care (the fifth-largest area difference, Fig. 1d), domain 6: participation (the sixth-largest area of difference, Fig. 1g), and domain 1: cognition (the seventh-largest area of difference, Fig. 1b). Most of the general adult population in Taiwan was able to deal with their daily lives without any help or the use of assistance devices. In contrast, for adults with disabilities, assistive devices and help from others played an important role, especially in domain 3: self-care, domain 2: getting around, and domain 5: life activities (Fig. 1b-h).

In conclusion, the gaps in AP functioning scores between the general adult sample and the disabled adults were near the 60th percentile for all domains and in both dimensions. The largest disparity curve (the greatest difference in the percentile of people experiencing no problems in daily life) between the general adult population and adults with disabilities was in self-care. 30% of adults with disabilities had no problems in self-care, compared with 90% in the general adult population (gap difference: 60%). The second largest difference was getting around. This was 20% for adults with disabilities and 80% for the general adult population (gap difference: 60%). The third largest difference was in life activities. This was 10% for adults with disabilities and 80% for the general adult population (difference: 70%; Tables 4 and 5, Fig. 1b-h).

**Table 5:** The WHODAS 2.0 norms score of people with disabilities by gender and age group

Performance	All	Male				Female			
		18–39	40–64	≥65	All Male	18–39	40–64	≥65	All Female
n	144,850	12,106	35,609	30,216	77,931	8425	24,927	33,567	66,919
Mean ± SD	45.10 ± 25.54	32.79 ± 21.32	40.68 ± 24.32	51.11 ± 27.19	43.50 ± 25.91	34.49 ± 20.39	40.56 ± 21.87	54.83 ± 25.54	46.95 ± 24.97
Median	42.45	30.19	42.45	50	40.57	32.61	38.68	55.43	44.57
Range	0–100	0–100	0–100	0–100	0–100	0–100	0–100	0–100	0–100
10 percentile	13.04	6.60	9.43	14.13	10.38	9.43^b^	13.21^b^	19.57^b^	15.22^b^
20 percentile	21.70	13.21	18.48	23.91	19.57	16.04^b^	20.75^b^	30.43^b^	23.58^b^
30 percentile	28.30	19.81	25.47	33.70	26.42	21.74^b^	27.36^b^	39.13^b^	31.13^b^
40 percentile	35.85	25.47	32.08	42.39	33.96	27.36^b^	33.02^b^	47.17^b^	37.74^b^
50 percentile	42.45	30.19	38.68	50.00	40.57	32.61^b^	38.68	55.43^b^	44.57^b^
60 percentile	50.00	35.85	44.57^a^	58.70	48.11	37.74^b^	44.34	63.04^b^	51.89^b^
70 percentile	58.49	41.51	52.17^a^	68.48	56.60	43.40^b^	50.94	70.65^b^	60.38^b^
80 percentile	68.87	49.06	62.26^a^	78.26	67.39	50.94^b^	58.49	79.35^b^	70.65^b^
85 percentile	75.00	54.72	67.92^a^	83.70	73.91	55.66^b^	64.15	84.78^b^	76.09^b^
90 percentile	82.61	62.26	75.47^a^	89.13	82.08	62.26	71.70	90.22^b^	83.70^b^
95 percentile	91.51	73.58^a^	85.85^a^	96.23^a^	91.30	73.30	81.13	95.65	92.39^b^
100 percentile	100.00	100.00	100.00	100.00	100.00	100.00	100.00	100.00	100.00
Capability	All	Male				Female			
		18–39	40–64	≥65	All Male	18–39	40–64	≥65	All Female
n	144,850	12,106	35,609	30,216	77,931	8425	24,927	33,567	66,919
Mean ± SD	51.12 ± 27.46	35.59 ± 22.81	45.26 ± 26.20	59.06 ± 28.24	49.11 ± 27.88	37.13 ± 21.71	45.10 ± 23.54	63.78 ± 25.94	53.46 ± 26.76
Median	49.06	33.02	42.45	61.32	46.74	34.91	42.45	67.39	51.89
Range	0–100	0–100	0–100	0–100	0–100	0–100	0–100	0–100	0–100
10 percentile	15.09	7.55	11.96	18.48	13.04	10.38^b^	16.04^b^	25^b^	17.92^b^
20 percentile	24.53	15.09	20.74	30.43	21.74	17.92^b^	23.58^b^	38.68^b^	27.36^b^
30 percentile	33.02	21.70	28.30	41.30	30.19	23.58^b^	30.43^b^	49.06^b^	35.85^b^
40 percentile	40.57	27.36	35.85	51.89	38.68	29.25^b^	36.79^b^	58.70^b^	43.48^b^
50 percentile	49.06	33.02	42.45	61.32	46.74	34.91^b^	42.45	67.39^b^	51.89^b^
60 percentile	58.49	38.68	50.00^a^	70.75	55.66	39.62^b^	49.06	75.47^b^	60.87^b^
70 percentile	67.92	44.34	59.43	80.19	66.04	46.23^b^	56.60	82.61^b^	70.75^b^
80 percentile	79.35	53.77	69.81^a^	88.04	77.36	54.72^b^	66.04	89.62^b^	81.13^b^
85 percentile	85.85	59.43	76.42^a^	92.39	83.96	60.38^b^	71.70	92.45^b^	86.79^b^
90 percentile	91.30	67.92	83.96^a^	95.65	90.57	67.92	79.25	95.65	91.51^b^
95 percentile	96.74	80.19^a^	93.48^a^	99.06	96.74	79.25	89.13	99.06	96.74
100 percentile	100.00	100.00	100.00	100.00	100.00	100.00	100.00	100.00	100.00

^a^Male Scores > Female Scores; ^b^Female Scores > Male Scores

## Discussions

The present study was the first normative value study in Taiwan using WHODAS 2.0 and a face-to-face survey approach. This study provides a benchmark of the WHODAS 2.0 and may have consequences for the application of welfare services and budget allocation. Having an overview of how adults with disabilities function in comparison to the general adult population is important to better inform and guide the disability evaluation system in the future. By understanding the differences between similar studies, our findings can also provide an evidence base for decision makers in clinical and population settings, and with respect to related health issues.

Our AP functioning norm study was comparable in size to other multi-country studies aimed at developing AP functioning norms, which had interview samples sizes between 1000 and 1500 [17]. Although we only surveyed participants from two cities in the present study, we found no significant difference between the two general adult population samples we used. Caution should be taken in applying these results or comparing them with other populations.

### Population norms in Taiwan

Health assessments vary in their data collection methods, using assessments such as the SF36, EQ-5D, quality of life, and WHODAS 2.0. Most normative studies conducted in other countries collect data either face-to-face [4, 6, 11] or by telephone [1, 3, 8]. The present study is the first, in both Taiwan and Asia, to develop an AP functioning norm for a general adult population using a face-to-face interview method. It is essential to assess the population AP functioning norm to facilitate the monitoring of many health policy interventions, for instance the general health screening and exams provided by the NHI in Taiwan. Because of this, our analysis was based on the age cut-points used in the health exams provided by the NHI. Moreover, norms are required to be a mirror of the general population, and should therefore include disabled and other vulnerable groups. Our findings are potentially a valuable reference, which can be used for comparison and application in other studies.

Although, we still had missing data with our samples in the AP norm of population, we only excluded 8 cases and the remainders missing rate was handful for whole data. Furthermore the remainder’s missing items were imputed by their mean score of every domain so the results were reliable.

On the whole, we found that the general adult population of Taiwan had less limitation in AP functioning and was healthier than the general population reported in the multiple-countries survey used for comparison in this study [17]. This discrepancy may be due to the language barrier between countries in the multiple-countries survey. The collection methods were diverse and thus linguistic nuances may have affected the measurement of AP. Another reason could be that the physical and social environmental factors were not adjusted in all of the studies that compared AP functioning to a normative value. Although some environmental factors may not have been considered, we assume that variation due to random factors exists in all countries. The most important contribution of the present study was the development of the AP functioning normative value. This enables exploration of the quality of life and examination of the functional gap between the global population, the general adult population in Taiwan and the disabled adult population in Taiwan.

### Comparison of the general adult population and adults with disabilities in Taiwan

The FUNDES-Adult, which is based on WHODAS 2.0, is part of an assessment used by the Taiwan disabilities eligibility system. Thus, it is essential to understand the distribution of AP functioning, as represented by a normative value, to allow comparison with other studies. Based on our findings, the AP curve increased rapidly in the lowest percentiles of the disabled population. Roughly 90% of the general adult sample scored under score 10, whereas only 10% of the disabled population scored under 10 in most domains (Fig. 1b-h). These findings show that there was a large difference in the capability element of AP functioning between the general adult and disabled adult populations.

With regards to differences in capability and performance scores for adults with disabilities, we found that assistive devices and help from others were important factors. This is especially the case for the domains of self-care, getting around, and life activities. To better facilitate the implementation of policy on disability, it is critical to have timely provision of assistive devices, and to increase physical environment access through universal design. Further, it is important to promote policies based on the Convention on the Rights of Persons with Disabilities (CRPD), which could increase the participation of people with disabilities [27]. These steps may ultimately reduce the AP functioning gap between the general adult population and adults with disabilities. Our findings will also help the Taiwanese government to predict the requisite welfare resources and allocate them in advance, based on the AP functioning gap in different domains.

However, it is important to use caution when discussing the gap in functional scores between two groups. In the present study, the age distribution for the disabled adult sample was greater than for the general population. Thus, caution should be used when comparing between these two groups with regards to employment rates and retirement. Future research should make further adjustments for age before engaging in more in-depth discussion of the outcomes.

### Reducing the gap in AP functioning between the general adult population and adults with disabilities

People in Taiwan appear to be similar to other populations across the globe. Most Taiwanese adults (up to the 70th percentile) conduct their daily lives without any help from others or the use of assistive devices. About 90% of adults with disabilities, however, do require such help. This outcome implies there is a huge AP functioning gap between the general adult population and adults with disability in both performance and capability. Further, it implies that adults with disabilities have reduced quality-of-life, greater barriers, and limited circumstances, even if they have an assistive device or another person to assist them.

A core value of long-term care for those with disabilities is to maintain the individual’s independence and to ensure their right to participate or to act in their social roles. Reducing the gap in their AP functioning is key to achieving these goals, and doing so can provide an index representing the advancement of human rights and a universal concept for disability. Based on the ICF, a multidimensional approach is needed to reduce the gap in AP functioning. These approaches include health promotion, a friendly and supportive environment, timely social welfare services and delivery systems, and integrated policy.

It is a difficult challenge for both the government and the people of Taiwan to establish and discuss in advance how to best apply resources for social welfare. If research can categorize disabilities based on functioning, and identify when people are likely to have multiple disability types and require different types of assistance, then the government can effectively allocate sufficient resources and budget in advance. The results should be useful to the government when formulating health policy. Despite the fact that the samples in present study were not taken from across the whole country, we found no differences in sex, age group, location, or proportion of adults with disabilities between our two population samples. We conclude, therefore, that these samples are reasonably representative of the Taiwanese population in general.

## Conclusions

This is the first study to develop an AP population normative value using face-to-face survey methods in Taiwan. It will allow members of the public to assess their health status in relation to the average health status of the population. The study also provides a benchmark to compare the health status of the general population of Taiwan with the results of other studies and with that of adults with disabilities in Taiwan. In addition, our results also illustrate the AP functioning gap between general adults and adults with disabilities. Reducing the gap in AP functioning thus represents a big challenge, both for the government and for the whole population of Taiwan. The results also highlight the critical importance of advance allocation of resources for social welfare services to different sectors of the population. Thus, the present study provides a valuable evidence base for health policy decision making, and a reference for NDEDS modification.

## Abbreviations

AP: Activities and participation
CRPD: Convention on the Rights of Persons with Disabilities
ICF: International Classification of Functioning, Disability and Health
IRT: Item-response-theory
MCSS: Multi-Country Survey Study
NDEDS: National Disability Eligibility Determination System
NHI: National Health Insurance
WHODAS 2.0: World Health Organization Disability Assessment Schedule 2.0
